# Risk Factors for Childhood Overweight and Obesity in Ukraine and Germany

**DOI:** 10.4274/jcrpe.galenos.2019.2018.0157

**Published:** 2019-09-03

**Authors:** Vira Yakovenko, Laura Henn, Markus Bettendorf, Natalia Zelinska, Galyna Soloviova, Georg F. Hoffmann, Juergen Grulich-Henn

**Affiliations:** 1 Ruprecht-Karls-University Heidelberg, University Children’s Hospital, Department of General Pediatrics, Heidelberg, Germany; 2 Otto-von-Guericke University Magdeburg, Institute of Psychology, Magdeburg, Germany; 3 Ukrainian Center of Endocrine Surgery and Transplantation of Endocrine Organs and Tissues, Kiev, Ukraine; 4 Ukrainian Children Specialized Hospital “OHMATDIT”, Kiev, Ukraine

**Keywords:** Obesity, risk factors, childhood, adolescent, overweight

## Abstract

**Objective:**

The prevalence of overweight and obesity in childhood and adolescence are rapidly increasing and influenced by genetic, familial, environmental, socioeconomic and cultural factors. The aim of the study was to compare risk factors for childhood obesity in Ukraine (UA) and Germany (DE) using comparable investigative tools.

**Methods:**

Two groups of children, aged 8 to 18 years, from DE (93 children) and UA (95 children) were divided into overweight and obese groups. Anthropometric data and detailed medical history were collected.

**Results:**

Risk factors in pregnancy (prematurity, weight gain >20 kg, early contractions) were equally frequent in both groups. Positive correlations of body mass index (BMI)-standard deviation score (SDS) between children and mothers were noted. The proportion of family members with diabetes mellitus was lower in the UA group. Obesity was more frequent at one year of age in DE children. The DE group also became overweight at an earlier age and remained overweight over a longer period of time compared to UA. The mean BMI-SDS of obese children was lower in the UA group. In both groups waist circumference to height ratio was >0.5, indicating presence of a cardiometabolic risk factor. About half of the patients in both groups had blood pressure values exceeding the 95^th^ percentile.

**Conclusion:**

Similar risk factors for obesity were observed among two groups of children in UA and DE. Differences were observed regarding the prevalence of specific risk factors for childhood obesity. Population-specific distribution of risk factors needs to be considered in order to optimize prevention and treatment strategies.

**What is already known on this topic?**

Obesity in children and adolescents has become an increasing and widely-distributed health problem, especially in industrialized countries. Risk factors include both genetic predisposition and socioeconomic factors. Only a few studies have compared the distribution of socioeconomic risk factors for childhood obesity across different countries.

**What this study adds?**

The current study is the first one to analyze the distribution of risk factors for obesity in Ukraine and Germany. Similar risk factors for obesity were observed in both countries although the prevalence of these risk factors varied between the two populations.

## Introduction

Obesity in children and adolescents has become an increasing and widespread health problem, especially in industrialized countries. According to the World Health Organization (WHO) more than 41 million children under the age of five suffered from overweight or obesity in 2016 (1). In Germany (DE), according to two large national studies (KIGGS and Crescnet), 15-17% of children aged three to 17 years were overweight and 6.2-7.6% were obese (2). In Ukraine (UA), the incidence of childhood obesity until recently was much lower but has shown a significant rise over the last decade, increasing from 0.083% among age groups 0-18 years in 2003, to 1.23% in 2009 and 1.34 in 2016 (3,4).

Numerous risk factors for childhood obesity have been discussed in the literature. Parental overweight is considered to be a particularly important indicator for overweight and obesity among their children (5). Further risk factors include characteristics of the maternal medical history during pregnancy and the perinatal period. Thus, excessive maternal weight gain during pregnancy and maternal smoking during pregnancy contribute to the development of obesity and its incidence (6). In addition, high birth weight constitutes a significant risk factor for the development of childhood obesity (7). Moreover, children with rapid weight gain during the first four to six months of life have been shown to have an increased risk of developing obesity by the age of seven years. Breastfeeding over a period of at least six months reduces the risk of obesity by 40% (8). Children who have become overweight by the age of six years often remain overweight at age 14 years (9).

Since obviously both genetic and socioeconomic factors have an influence on the development of childhood obesity, we investigated similarities and differences regarding these risk factors in two cohorts from two different European countries (UA and DE).

## Methods

The study was conducted at two university children hospital outpatient centers, in Simferopol, UA (2010-2011) and in Heidelberg, DE (2012-2013). At both centers WHO standards were used for definition of overweight and obesity. Inclusion criteria were: age 8-18 years, BMI 85^th^-95^th^ percentile (overweight) and >95^th^ percentile (obesity), informed consent. Exclusion criteria were: age <8 years or >18 years, chronic endocrinopathies (diabetes mellitus ect.), genetic disorders, disabled children, chronic inflammatory diseases (e.g. M. Crohn), lack of informed consent. Inclusion and exclusion criteria were identical. Comparable questionnaires for risk factors and for both patient and family medical history were used at both centers.

The study population in the UA consisted of 95 (35 girls and 60 boys) otherwise healthy children aged 10 to 18 [mean±standard deviation (SD)=13.5±0.4] years, divided into an overweight (36 children) and an obesity (59 children) group. The study population in DE consisted of 93 (46 girl, 47 boys) otherwise healthy children aged 8 to 18 years (mean±SD=12.5±2.9) years divided into an overweight (24 children) and an obesity (69 children) group.

The populations were comparable according to sex, Tanner stages and age and did not show any statistically significant difference. Physical examination in outpatient departments included measurement of height, weight (in underwear), waist and hip circumference, blood pressure and examination of the skin for acanthosis nigricans. BMI-SDS, waist/hip circumference ratio (WHR), and waist circumference/height ratio (WHtR) were calculated. Standardized patient history included pregnancy history (maternal obesity, weight gain of more than 20 kg, arterial hypertension, premature (before 37^th^ gestational week) or postmature (after 42^nd^ gestational week) delivery, perinatal asphyxia, duration of breastfeeding and family history (overweight or obesity, diabetes mellitus, arterial hypertension in first degree relatives). All risk factors were recorded with regard to their presence or absence. The children’s history of weight gain was also evaluated (reported by parents and according to medical reports).

The study was approved by the Ethics Committee of the Medical Faculty of the University of Heidelberg (approval number: S-337/2013, approval date: 22/07/2013). Written consent was taken from the parents at the beginning of the study in accordance with the Declaration of Helsinki.

### Statistical Analysis

Statistical analysis was performed using Statistical Package for the Social Sciences (SPSS), version 20.0.0 for Windows (SPSS Inc., Chicago, IL., USA). As part of the descriptive analysis, sample size, arithmetic mean, median, maximum (max) and minimum (min), and SD were determined. To test the variables for normality of the distribution, the Kolmogorov-Smirnov test with an error probability of 0.05 was used. The t-test and the Mann-Whitney U test were used to test for significant differences between groups. Gender differences in categorical variables were tested with the chi-square test. A statistical parameter of p<0.05 was considered significant.

## Results

The risk factors during pregnancy occurred with an approximately equal frequency among the DE and UA populations (Table 1). Premature birth was reported in 8-12% of all children. In the DE population 21% of mothers of overweight children and 33% of mothers of obese children gained more than 20 kg during pregnancy. In the UA population the incidence of excessive weight gain during pregnancy among mothers of obese children was 25%. Early contractions were reported by 13-17% (DE) and 20-28% (UA) of mothers, respectively (Table 1).

The prevalence of obesity was significantly higher in first-degree relatives of obese children compared to relatives of overweight children (p<0.05). A highly significant positive correlation was found between the children’s and the mothers’ BMI-SDS (1.03±1.26 for the overweight and 1.96±1.20 for the obese group) in the DE population (r=0.46, p<0.0001).

Birth weight (3295±474 g in UA population, 3352±517 g in DE population) and birth length (50.6±2.4 cm in UA population, 51.1±2.1 in DE population) as well as their relation to BMI-SDS did not differ among the two groups. In both populations, obese children were more frequently obese at the age of one year (33.3% in UA population, 27.3% in DE population) compared to overweight children (14.3% in UA population, 17.4% in DE population). On average, the children in the UA population were breastfed for a longer period of time (6.8±6.7 months in the obese, 7.1±7.2 months in the overweight group) compared to the children in the DE population (4.6±6.2 months in the obese, 6.1±7.3 months in the overweight group). The DE population became overweight significantly earlier and therefore remained overweight over a significantly longer period. The mean duration of obesity in the DE population was 7.6±4.3 years (min 1.2, max 18.0), the mean duration of overweight 7.2±5.0 years (min 1.1, max 18.0) compared to 5.7±3.5 years (min 1.0, max 13.7) and 4.71±3.5 years (min 0.8, max 14.0), respectively in the UA population. In both populations, the duration of the overweight period significantly influenced the BMI-SDS. The mean BMI-SDS of obese children was lower in the UA population than in those in the DE population [BMI-SDS (UA) 2.31±0.49 *vs* BMI-SDS (DE) 2.52±0.55 (p<0.05)]. There were no significant differences in BMI-SDS in overweight children. In both populations, the BMI-SDS was significantly influenced by the Tanner stage (p<0.05) (Table 2).

There were significant differences between the DE and UA populations regarding WHR and WHtR (p<0.05) (Table 3). The children in the UA population showed no significant differences in the WHR according to sex or age compared to the DE population. In the DE population the WHtR was significantly influenced by gender (p<0.05). In both populations children showed central trunk obesity (predominantly the girls in the UA population). In obese children the WHtR exceeded 0.5. In the DE population 54% of obese patients had blood pressure values above the 95^th^ percentile compared to 22% in the UA population (Table 3). Acanthosis nigricans was observed twice as often among patients in the DE population. In both populations, acanthosis nigricans was observed significantly more frequently in obese patients compared to the overweight patients (Table 3).

## Discussion

The greater the frequency of risk factors identified in mothers during pregnancy, the greater was the likelihood that the child’s BMI-SDS would be increased (10). Mothers with a normal BMI usually gain 11 to 16 kg during pregnancy (11). In the present study we found in both countries that an excessive weight gain of mothers during pregnancy was associated with the risk for childhood obesity (12,13).

The prevalence of birth before 37 or after 42 gestational weeks in the DE study population was within the expected range (14). These figures showed a somewhat higher prevalence in the UA group. Gestational hypertension was found to occur in 5-10% of all pregnancies (15). The same prevalence was observed in the mothers of overweight children in our study. By contrast, the mothers of children with obesity were twice as likely to have developed gestational hypertension (17%). However, this finding was limited to the DE population. There are no exact data on the prevalence of early contractions with estimates ranging from 5% up to 35% of pregnancies (16). If early contractions are considered as a potential threat of premature birth, the increased incidence may be considered as a relevant risk factor for childhood overweight and obesity. Perinatal asphyxia may be equally considered a risk factor. In our patients, the prevalence of perinatal asphyxia both in the UA and in the DE populations exceeded the prevalence of 0.5-1% (5-10:1000 births) observed in the general population (17).

Several studies have shown that the BMI-SDS of the parents plays an important role influencing the BMI-SDS of a child (18). In the current study, maternal BMI-SDS was significantly higher in obese children as compared to the values of mothers of overweight children. A highly significant positive correlation was found between the BMI-SDS of the children and the BMI-SDS of their parents. There was a significant positive correlation between the number of familial risk factors (diabetes mellitus and arterial hypertension) and the BMI-SDS values of the children.

The prevalence of arterial hypertension ranges from 32.3% in developed to 40.8% in developing countries (19). The present study indicates that first degree relatives of obese children have a higher prevalence of arterial hypertension and furthermore have a significantly higher prevalence of diabetes mellitus. According to the atlas of the International Diabetes Federation, the prevalence of diabetes in DE was 10.6% in 2015, with a proportion of undetected diabetes of 38.2%. In UA during the same year, the respective numbers were 8% and 43.2% (20). We suspect that in the UA population there may be a greater deficit in the diagnosis of DM and arterial hypertension in adults, and that the actual frequency is most likely significantly higher. This suspicion is supported by the increased incidence of obesity in families in the UA population.

Currently there are no studies showing the prevalence of obesity in one-year-old children. In the US, the prevalence among children aged 0-2 years was reported as high as 8.1% (21). In our study, the prevalence of overweight and obesity at the age of one year was much higher with 17-27% in the DE population and 14-44% for children in UA. The results support the potential importance of BMI in children under two years of age to identify an increased risk of later obesity (22). Furthermore, breastfeeding plays an important role in the prevention of obesity. Studies have shown that formula-fed infants have a higher chance of becoming obese later in life compared to breastfed infants (23).

So far only a small number of studies have investigated the onset of obesity. Most of these studies have identified the preschool age of 5-7 years as a risk period (24). In our study, children and parents reported significant weight gain starting from the age of 5-6 years in the DE population and 7-10 years in the UA population. This information might be important to identify the right timing for intervention, investigation and prevention. The increase in BMI-SDS is influenced by the duration of overweight and/or obesity in childhood. Therefore, initiating intervention and therapy as early as possible is important. The children of the UA population had lower BMI-SDS compared to the DE population. To our knowledge this is the first study comparing these two countries.

The data on which parameter is the better one to describe abdominal fat distribution in children is controversial. While American sources showed that BMI and WHtR did not differ in identifying children with cardiovascular risk factors, other studies found that waist circumference and WHtR were better predictors of cardiovascular risks compared to BMI (25). Recent research has shown that WHR may not be an informative parameter for cardiometabolic risk. On the other hand, WHtR is associated with cardiometabolic risk compared to BMI-SDS in both adults and children. In both populations children showed trunkal obesity. In German children aged 12-18 years WHR was reported as 0.83±0.05 in boys and as 0.78±0.06 in girls (26).

The normal value for WHtR has been reported to be below 0.5 (27). In our study, WHtR was >0.5 in children with obesity from the UA population and in children with overweight and with obesity from the DE population which may indirectly be interpreted as a cardiometabolic risk factor.

The prevalence of high blood pressure in obesity varies: from 21-35%, up to 46% in children and 40% in adults (28). According to other studies the prevalence of hypertension in overweight children increased from 6.6% in boys aged 2-5 years to 13.3% in adolescents 16-19 years; and in girls 4.4% and 16.3% respectively (29). In our study, we found a prevalence of 44.1% in the UA obese group while in the DE group the prevalence of hypertension was even higher, at 56.1%.

Acanthosis nigricans is associated with diabetes mellitus type 2 and insulin resistance and correlates strongly with obesity, although it has been reported to be present in 17% of healthy children (30). Acanthosis nigricans was observed twice as often in the DE patients compared to the UA population with a moderately higher prevalence compared to healthy children. In both populations, acanthosis nigricans was significantly more common in obese patients than in patients who were overweight.

### Study Limitations

The main limitation of our study is that we relied on self-reports of the parents for pregnancy history and history of weight gain of their children.

## Conclusion

The anamnestic risk factors for overweight and obesity in children were very similar in the DE and UA subjects, except for the number of familial risk factors which did not correlate with the BMI-SDS values in the UA population. We assume that in the UA population there is a greater deficit in the diagnosis of diabetes mellitus and arterial hypertension in adults and that a substantial fraction of adult cases of diabetes and arterial hypertension remain undiagnosed in the UA. The actual prevalence of these conditions is likely to be significantly higher. Relevant risk factors for the development of obesity include family and pregnancy history, as well as neonatal and infant medical history. Further important risk factors include anthropometric parameters. Since BMI normal values are age-dependend and increase during adolescents, BMI-SDS should be used for the evaluation of the degree of childhood obesity. WHtR, blood pressure and presence of acanthosis nigricans are important prognostic indicators for the risk of obesity related diseases, and should be determined in children and adolescents with overweight/obesity. The children from the DE population became overweight significantly earlier compared to the UA population. DE patients with obesity also had higher BMI SDS.

## Figures and Tables

**Table 1:**
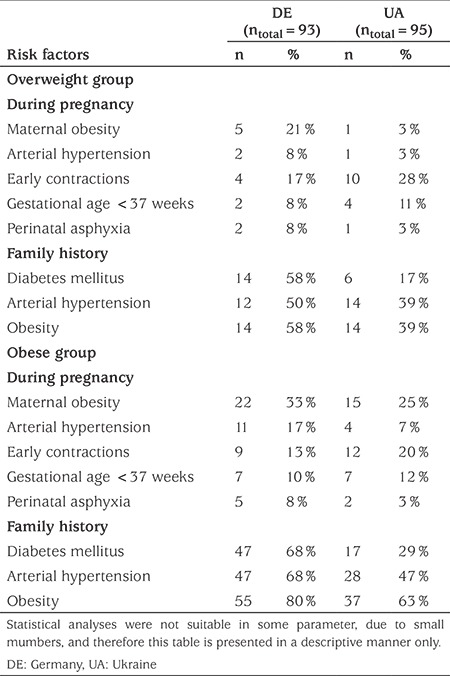
Prenatal and familial risk factors in the overweight and obese groups

**Table 2:**
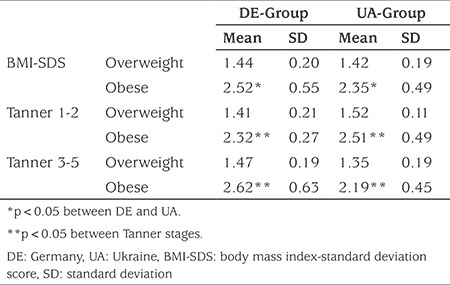
Anthropometric data

**Table 3:**
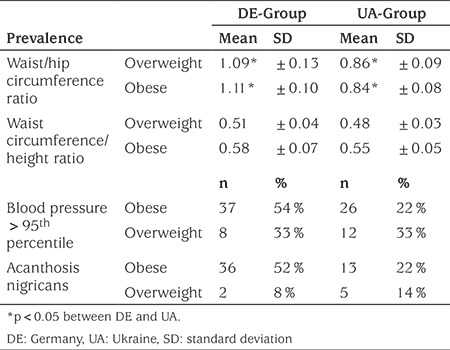
Risk factors for metabolic syndrome
